# Magnetic microbubble-mediated ultrasound-MRI registration based on robust optical flow model

**DOI:** 10.1186/1475-925X-14-S1-S14

**Published:** 2015-01-09

**Authors:** Mo Hou, Chunxiao Chen, Dalin Tang, Shouhua Luo, Fang Yang, Ning Gu

**Affiliations:** 1 Jiangsu Key Laboratory for Biomaterials and Devices, State Key Laboratory of Bioelectronics, School of Biological Science and Medical Engineering, Southeast University, Nanjing, 210096, China; 2 School of Computer Science and Technology, Jiangsu Normal University, Xuzhou, 221000, China; 3 College of Automation Engineering, Nanjing University of Aeronautics and Astronautics, 210016, Nanjing, China; 4 Mathematical Sciences Department, Worcester Polytechnic Institute, MA, 01609, USA

## Abstract

**Background:**

As a dual-modality contrast agent, magnetic microbubbles (MMBs) can not only improve contrast of ultrasound (US) image, but can also serve as a contrast agent of magnetic resonance image (MRI). With the help of MMBs, a new registration method between US image and MRI is presented.

**Methods:**

In this method, MMBs were used in both ultrasound and magnetic resonance imaging process to enhance the most important information of interest. In order to reduce the influence of the speckle noise to registration, semi-automatic segmentations of US image and MRI were carried out by using active contour model. After that, a robust optical flow model between US image segmentation (floating image) and MRI segmentation (reference image) was built, and the vector flow field was estimated by using the Coarse-to-fine Gaussian pyramid and graduated non-convexity (GNC) schemes.

**Results:**

Qualitative and quantitative analyses of multiple group comparison experiments showed that registration results using all methods tested in this paper without MMBs were unsatisfactory. On the contrary, the proposed method combined with MMBs led to the best registration results.

**Conclusion:**

The proposed algorithm combined with MMBs contends with larger deformation and performs well not only for local deformation but also for global deformation. The comparison experiments also demonstrated that ultrasound-MRI registration using the above-mentioned method might be a promising method for obtaining more accurate image information.

## Background

Compared with other medical imaging modalities, ultrasound imaging has been widely used in diagnosis and clinical applications owning to its merits of low-cost, real-time, high safety, and no documented side effects. By using proper contrast agents, the contrast and sensitivity of ultrasound imaging have been greatly improved [1,2]. However, ultrasound imaging is still limited because of the following reasons. Firstly, ultrasound is reflected very strongly when passing from tissue to gas, and vice versa. Secondly, the method is of limited use in diagnosing fractures because ultrasound does not pass well through bones. Finally, the quality of ultrasound imaging is mediocre when its contrast is lower than that of MRI or computed tomography (CT) [3,4].

Magnetic resonance imaging is another imaging tool which is non-invasive and capable of providing functional information with high spatial resolution and excellent soft-tissue contrast [5]. In particular, MRI can provide information about blood flow and vessel morphology and identify stenotic arteries for early treatment. Magnetic iron oxide nanoparticles with superparamagnetic property can be used as a powerful contrast agent for MRI to further increase its brightness and contrast. One shortcoming of MRI is that it can not provide real-time motion-related images. MRI can be affected by movement, making it unsuitable for investigating problems such as mouth tumors because coughing or swallowing can make the images less clear.

To sum up, different imaging modalities have their respective advantages and disadvantages in the spatial resolution, and no single imaging modality possesses all the advantages satisfying the need of all clinical applications. In many cases, US image and MRI are complimentary, and both modalities are needed to discern possible pathological changes in tissue [6]. Therefore, it is extremely desirable to fuse the image information of different modes. To fuse US and MRI together, US-MRI registration is required. Due to US image's strong noise, it is a great challenge to register US image with any other modality images. Some studies focused on three-dimensional US-MRI registration or three-dimensional US-CT registration in the field of operation navigation [7-12]. Whether feature-based or voxel-based registration is used, segmentation of multimodality medical images is required. The registration result depends directly on the segmentation results. It is fair to say that US image segmentation is a difficult issue at present.

We have carried out preliminary research on MRI-ultrasound registration based on dual modality contrast agent, namely MMBs, and have obtained some promising results [13,14]. Those elementary research results depend on how to select the region of interest (ROI) to certain extent, while running the registration code is time-consuming, and selecting ROI is a subjective process. To overcome the above limitations, a more efficient registration algorithm and semi-automatic segmentation of ROIs using MMBs are presented in this paper. With MMBs, the gray value within regions of interest (ROI) of US image and MRI is enhanced, which is very favorable for the segmentation to be performed. The MMBs discussed in the paper are the dual-modality contrast agent with both ultrasound and MRI contrast function. Gas filled microbubbles encapsulated with polymer, lipid or surfactant shells can be used as the most effective contrast agent for ultrasound imaging. Superparamagnetic iron oxide nanoparticles (SPIO) can be used as a powerful contrast agent for MRI. The combination of microbubbles and SPIOs, MMBs, can be used as the contrast agent for both US imaging and magnetic resonance imaging because the MMBs can overcome the shortcomings of magnetic nanoparticles or microbubbles, respectively. That is, the stability of microbubbles can be improved by embeding magnetic nanoparticles into the bubble shells. Moreover, the embedded nanoparticles can be delivered into desired regions under the guidance of magnetic field and can be released when suitable ultrasound exposure is chosen. Cai *et al *focused on the relationship between the MMBs structure and dual modality imaging, and gave a good overview on magnetic microbubbles for theranostics, including their preparation, imaging contrast agents (diagnostic) and drug delivery (therapeutic) [15].

Different from [15], the main contribution of this paper is the introduction of the above-mentioned dual modality contrast agent to multi-modality medical image registration. Using MMBs with the mean diameter of 3.98 μm prepared as described by Yang *et al *[2,6,16], this paper carries out the registration between US image and MRI, and comes to a conclusion that with the use of MMBs, the proposed algorithm performs well not only for global deformation but also for local deformation. The remainder of the paper is organized as follows: the registration method based on robust optical flow model between US image segmentation and MRI segmentation is described in Section 2. Section 3 provides several groups of comparison experiments, and analyzes the experimental results, while Section 4 concludes our paper.

## Methods

**Figure 1:**
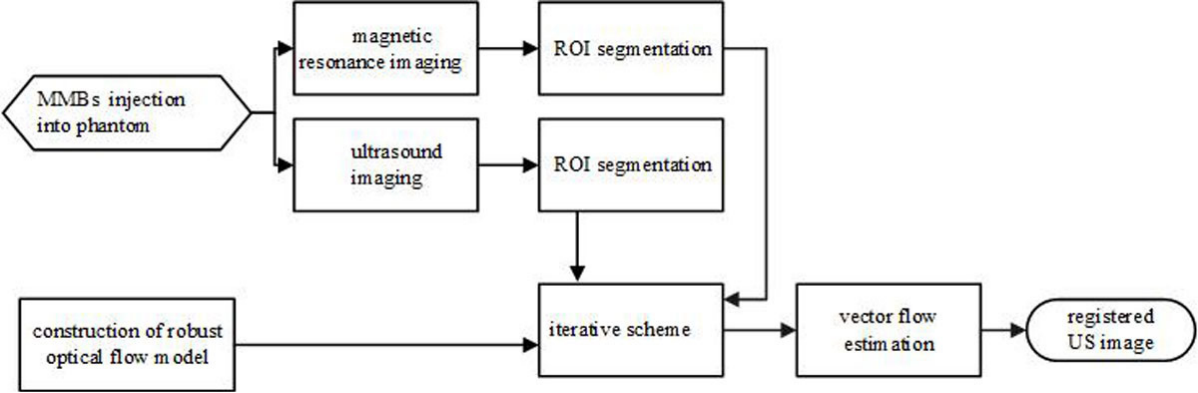
**Flow chart of the proposed MRI-US registration system based on MMBs and robust optical flow model**.

**Figure 2:**
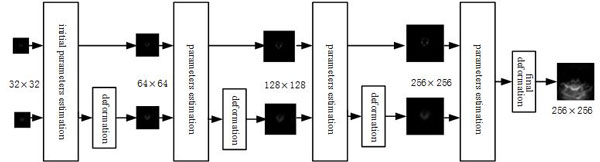
**Flow chart of Gaussian pyramid algorithm**.

As shown in Figure 1, MMBs were used in both ultrasound and magnetic resonance imaging process. In order to reduce the influence of the speckle noise on registration, semi-automatic segmentations of US image and MRI were carried out by active contour model. And then, a robust optical flow model between US image segmentation (floating image) and MRI segmentation (reference image) was built, and the vector flow field was estimated by the Coarse-to-fine Gaussian pyramid (see Figure 2) and graduated non-convexity (GNC) schemes. The registration method based on image intensity that directly uses gray information instead of the extraction feature process is widely concerned and quickly developed [17]. Image segmentation combined with optical flow algorithm can not only weaken the influence of noise, but also avoid feature extraction.

### Image segmentation with active contour model

Because all US images are confounded by speckle noise, fully automatic segmentation of US image is currently impossible. Therefore, the focus has been on semi-automatic techniques, particularly active contour models which can detect objects whose boundaries are not necessarily defined by gradient. Active contour models are used to evolve a curve, subject to constraints from a given image under a number of external and internal forces [18-22]. The external forces attract the curve to regions of interest in the image, whereas the internal forces hold the curve smooth. If initialized close to a boundary, the curve deforms to "hug" the boundary along its length, providing further adaptability to noise.

Assuming the evolving curve is defined as *C*, it will move through the spatial domain of the given image *I*_0 _to minimize the following energy function:

(1) $$\begin{array}{c} F\left(c_{1},c_{2},C\right)=\mu \cdot \text{Length}\;\;\left(C\right)+\nu \cdot \text{Area}\;\;\left(inside\;\left(C\right)\right)+\lambda_{11}\iint_{\;inside\;\left(C\right)}{\left|I_{0}\left(x,y\right)-c_{1}\right|}^{2}dxdy\;\\ \;\;\;\;\;\;\;\;\;\;\;\;\;\;\;\;\;\;\;+\lambda_{22}\iint_{\;outside\;\left(C\right)}{\left|I_{0}\left(x,y\right)-c_{2}\right|}^{2}dxdy. \end{array}$$

here *μ *≥ 0, *ν *≥ 0, *λ*_11 _> 0, and λ_22 _> 0 are fixed parameters, *I*_0_(*x*, *y*) is image intensity at pixel location (*x*, *y*), and the constants *c*_1_, *c*_2 _(depending on *C*) are the averages of *I*_0 _inside *C *and outside *C*, respectively. The length of the curve, **Length **(*C*), and the area of the region inside *C*, Area (*inside *(*C*)), are two regularizing terms. If the constant *μ *is larger, then only larger objects are detected, or objects that are grouped together. If it is small, then smaller objects will be detected. We do not want different objects close to each other to be interpreted as a single object. This is the reason we decided to set *μ *= 0 in Eq. (1). The minimization procedure uses iterative method and differential calculus. After initializing a curve close to the object boundary, the active contour model starts deforming to fit the local minimum so as to move towards the desired boundary and finally settles on it.

### Robust optical flow model construction for registration

Let *I*_1_, *I*_2 _denote the reference and floating images, namely MRI and US image. We need to determine the optical flow field (**u**, **v**) (the horizontal and vertical vectors) between *I*_1 _and *I*_2_, which represents the displacement between the above two images. The classical optical flow objective function in its spatially discrete form is written as

(2) $$\begin{array}{c} E\left(u\text{,}v\right)=\underset{i,j}{\sum }\{\rho_{D}\;\left[I_{1}\left(i,j\right)-I_{2}\left(i+u_{i,j},j+v_{i,j}\right)\right]+\lambda [\rho_{S}\;\left(u_{i,j}-u_{i+1,j}\right)+\rho_{S}\;\left(u_{i,j}-u_{i,j+1}\right)\\ \begin{array}{cccc} & & & \begin{array}{ccc} & & +\rho_{S}\;\left(v_{i,j}-v_{i+1,j}\right)+\rho_{S}\;\left(v_{i,j}-v_{i,j+1})]\right\} \end{array} \end{array} \end{array}$$

Where *ρ***_D _**and *ρ***_S _**are penalty functions, and *λ *is a regularization parameter. Optical flow method is sensitive to noises because it is on the basis of differential technology, and some filters (both high-pass and low-pass) are used to reduce this bad effect [23]. After every iteration median filtering the intermediate optical flow field can effectively denoise the intermediate flow field, prevent gross outliers, and make non-robust methods more robust [24]. When the median filter is used to optimize the objective, it will lead to lower energies. Especially, the optimization of the classical model, with interleaved median filtering and an auxiliary flow field $\text{(}\hat{u}\text{,}\hat{v}\text{)}$[25,26], approximately minimizes

(3) $$\begin{array}{l} E(u\text{,}v\text{,}\hat{u}\text{,}\hat{v})=\sum_{i,j}\left\{\rho_{D}\;[I_{1}(i,j)-I_{2}(i+u_{i,j},j+v_{i,j})]+\lambda_{1}[\rho_{S}\;(u_{i,j}-u_{i+1,j})+\rho_{S}\;(u_{i,j}-u_{i,j+1}\right)\\ \begin{array}{cccc} & & & \begin{array}{ccc} & & +\rho_{S}\;(v_{i,j}-v_{i+1,j})+\rho_{S}\;(v_{i,j}-v_{i,j+1}) \end{array} \end{array}]\}+\lambda_{2}\;({\left\|u-\hat{u}\right\|}^{2}+{\left\|v-\hat{v}\right\|}^{2})\\ \;+\lambda_{3}\sum_{i,j}\sum_{(i^{'},j^{'})\in N_{i,j}}(\left|{\hat{u}}_{i,j}-{\hat{u}}_{i^{'},j^{'}}\right|+\left|{\hat{v}}_{i,j}-{\hat{v}}_{i^{'},j^{'}}\right|)\;. \end{array}$$

where λ_1 _is a regularization parameter, λ_2 _and λ_3 _are scalar weight, and N_i,j _is the set of neighbor pixels of pixel (i,j). By alternately minimizing (4) and (5), we can optimize the objective function as in (3):

(4) $$\begin{array}{l} E(u\text{,}v)=\sum_{i,j}\left\{\rho_{D}\;[I_{1}(i,j)-I_{2}(i+u_{i,j},j+v_{i,j})]+\lambda_{1}\;[\rho_{S}\;(u_{i,j}-u_{i+1,j})+\rho_{S}\;(u_{i,j}-u_{i,j+1}\right)\\ \begin{array}{cccc} & & & \begin{array}{ccc} & & +\rho_{S}\;(v_{i,j}-v_{i+1,j})+\rho_{S}\;(v_{i,j}-v_{i,j+1}) \end{array} \end{array}]\}+\lambda_{2}\;({\left\|u-\hat{u}\right\|}^{2}+{\left\|v-\hat{v}\right\|}^{2})\;. \end{array}$$

(5) $$E\left(\hat{u}\text{,}\hat{v}\right)=\lambda_{2}\;\left({∥u-\hat{u}∥}^{2}+{∥v-\hat{v}∥}^{2}\right)+\lambda_{3}\;\underset{i,j}{\sum }\underset{\;\left(i^{\prime },j^{\prime }\right)\in N_{i,j}}{\sum }\left(\left|{\hat{u}}_{i,j}-{\hat{u}}_{i^{\prime },j^{\prime }}\right|+\left|{\hat{v}}_{i,j}-{\hat{v}}_{i^{\prime },j^{\prime }}\right|\right)\;.$$

By using the alternating optimization strategy, with $\text{(}\hat{u}\text{,}\hat{v}\text{)}$ fixed, we minimize (4) with regard to (**u**, **v**); with (**u**, **v**) fixed, we minimize (5) with regard to $\text{(}\hat{u}\text{,}\hat{v}\text{)}$

(6) $${û}_{i,j}^{\;\;\left(k+1\right)}=median\;\;\;\left(Neighbors{\;}^{\text{(}k\text{)}\;}\cup \;Data\;\;\right)$$

where $Data\;\;=\left\{u_{i,j},\;u_{i,j}\pm \frac{\lambda_{3}}{\lambda_{2}},\;u_{i,j}\pm \frac{2\lambda_{3}}{\lambda_{2}}\;\ldots ,\;u_{i,j}\pm \frac{\left|N_{i,j}\right|\lambda_{3}}{2\lambda_{2}}\;\right\}$,

$Neighbors{\;}^{\;\text{(}k\text{)}}=\left\{\;{û}_{i^{\prime },j^{\prime }}^{\;\left(k\right)}\;\right\}$ for (*i*^'^, *j*^'^) ∈ *N*_*i,j *_and ${û}^{\left(0\right)}=u$.

for ${\hat{v}}_{i,j}^{\;\;\left(k+1\right)}$, its proof is similar with ${û}_{i,j}^{\;\;\left(k+1\right)}$. Coarse-to-fine Gaussian pyramid and graduated non-convexity (GNC) schemes are adopted to estimate (**u**, **v**) and $\text{(}\hat{u}\text{,}\hat{v}\text{)}$[24]. A two stage GNC process is adopted and 3 warping steps per pyramid level are performed. After every warping step, (**u**, **v**) are set to be $\text{(}\hat{u}\text{,}\hat{v}\text{)}$. Finally, $\text{(}\hat{u}\text{,}\hat{v}\text{)}$ are taken as the final flow vector field estimate. The framework of Gaussian pyramid algorithm is shown in Figure 2, and the penalty functions are set according to Sun et al [25].

## Results & discussion

### Materials and data acquisition

#### Dual modality contrast agent

MMBs were obtained from Jiangsu Laboratory for Biomaterials and Devices. MMBs can increase magnetic resonance susceptibility, and negatively enhance T2-weighted (T2*WI) imaging signal, namely, decrease the gray value of T2*WI imaging. They can also give strong ultrasound backscattering echo intensity and positively increase the brightness of US image.

#### Phantom

Phantom was made from glycerol, agar and water ratio of 3:4:90, and produced by Jiangsu Laboratory for Biomaterials and Devices, in which a "U" shaped silicone tube with external diameter of 9 mm and inner diameter of 7 mm is "vertically" sitting in the agar phantom. Three fatty objects which size is about a diameter of 5 mm were laid in the tube. When US imaging or magnetic resonance imaging with MMBs was carried on, a solution (0.1 g/ml) containing MMBs was injected into the silicone tube; while when US imaging or MRI without MMBs was carried on, purified water was injected into the silicone tube. Regardless of whatever MMBs is used, fluid in the tube remains stable during imaging.

#### Experimental computing platform

Our algorithm implementation is based on the compatible personal computer installed MATLAB 2008b. In some cases, C++ language compilation system was also needed to perform some functions, so it is necessary to install VISUAL C++ 6.0 (or VISUAL C++ 2000.NET) on the above platform.

The three objects were imaged using the Ultrasonic imaging system of the GE LOGIQ3 PRO scanner (GE Medical System, USA) with a 4 MHz ultrasound transducer used as a transmitter as well as a receiver. B-mode US images were acquired with the instrument parameters (Gn 20; E/A 1/2; DR 78; AO 100%). T2*WI imaging of these objects was carried out with a clinical 0.3 T magnetic resonance imager (AIRIS II, Hitachi Ltd, JAPAN). Images were aquired with a matrix size of 256 × 256, field of view of 20 × 20 cm^2^, repetition time of 400 ms, section thickness of 4 cm, and echo time of 15 ms. The reasons the usage of 0.3 T magnetic resonance imager was as follows. Firstly, we had carried out multiple group comparison tests of magnetic resonance imaging using 1.0 T or above equipments based on MMBs, and obtained good imaging result. Secondly, now SIEMENS 0.3 T magnetic resonance imager was still used widely especially in underdeveloped area because of magnetic resonance imager's expensive upgrades.

**Figure 3:**
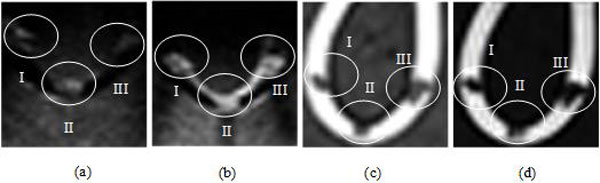
**Enhancement imaging based on MMBs**. **(a) **US image without MMBs. **(b) **US image with MMBs. **(c) **MRI without MMBs. **(d) **MRI with MMBs.

Figure 3 includes US and MRI images showing the effectiveness of MMBs. I, II and III in Figures 3(a)~(d) represent the above-mentioned three objects imaging. Without MMBs the tube boundary can't be seen, while with MMBs, the tube upper boundary and three targets can be seen from the B-mode image. Under ultrasonic conditions, gas imaging shows strong echo whereas liquid imaging has no echo, and MMBs almost float upward to the three targets and upper boundary of the tube, therefore the echo of the tube upper boundary and three targets is strong. The phantom surface is hard, and not easily deformed, and moreover the convex array probe is used, which lead to the poor contact between the probe and the phantom surface, and further bring lateral wall echo drop-out.

Compared with Figure 3(a), the brighter objects of ROI can be seen distinctly in Figure 3(b). This illustrates qualitatively that MMBs can positively enhance the intensity of US. Similarly, the MRIs of the tube without and with MMBs were shown in Figure 3(c)&(d), respectively. The result indicates that MMBs can significantly reduce T2*WI signal intensity. Certainly, we can quantitatively measure objects brightness of US images and MRIs to determine how much MMBs influenced US imaging and magnetic resonance imaging [2].

### Semi-segmentation of US images and MRIs

**Figure 4:**
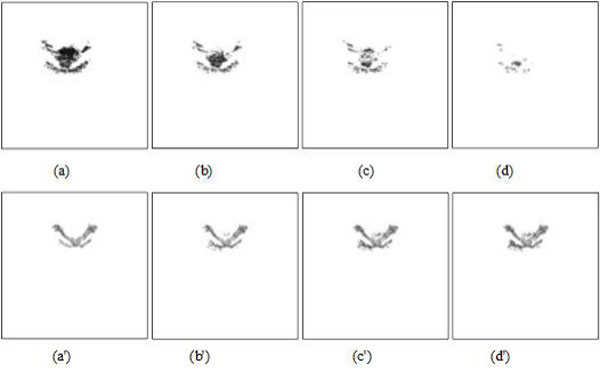
**US ROI segmentations by different values of parameter *ν *(*λ*_11 _= *λ*_22 _= 1, *μ *= 0) with active contour model**. Top: segmentation results without MMBs **(a) ***ν *= 100. **(b) ***ν *= 300. **(c) ***ν *= 500. **(d) ***ν *= 2000. Bottom: segmentation results with MMBs (*λ*_1 _= *λ*_2 _= 1, *μ *= 0) **(a') ***ν *= 2000. **(b') ***ν *= 500. **(c') ***ν *= 100. **(d') ***ν *= 5.

In Figure 4, when *λ*_11 _and *λ*_22 _are fixed to 1, and *μ *is fixed to 0, the segmentation results were compared with the change of parameter *ν*. The best segmentation was achieved when *ν *is set to 100 without MMBs. In contrast, we obtained the best segmentation when *ν *is set to 2000 with MMBs.

**Figure 5:**
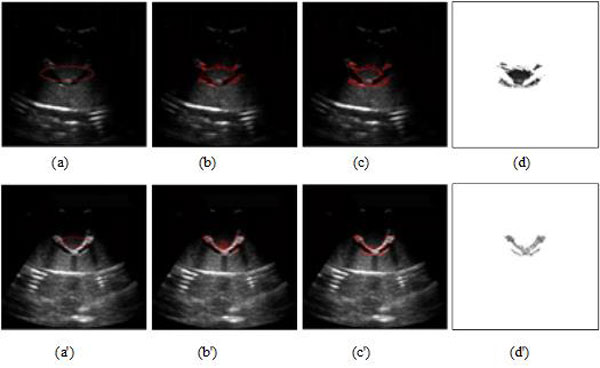
**Iterative segmentation process of US image with active contour model**. Top: results without MMBs **(a) **initialization. **(b) **20 iterations of US image. **(c) **50 iterations of US image. **(d) **segmentation result. Bottom: results with MMBs **(a') **initialization. **(b') **20 iterations of US image. **(c') **50 iterations of US image. **(d') **segmentation result.

Figures 5(a)~(d) and 5(a')~(d') show iterative segmentation process of US image with active contour model (λ_11 _= λ_22 _= 1, μ = 0) when *ν *is set to 100 without MMBs, and 2000 with MMBs, respectively. Compared with Figure 5(d) and Figure 5(d'), Figure 5(d') is the better segmentation result. The fundamental reason that the segmentation results have such a huge difference is the better contrast and brightness of the US image using MMBs than that not using MMBs, which is beneficial to segmentation of US image.

**Figure 6:**
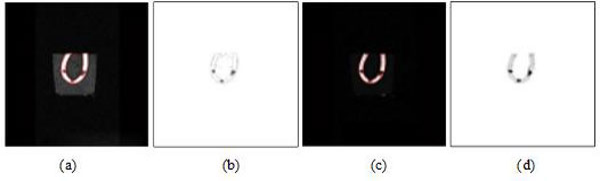
**Iterative segmentation process of MRI with active contour model**. **(a) **50 iterations of MRI without MMBs. **(b) **segmentation result of MRI without MMBs. **(c) **50 iterations of MRI with MMBs. **(d) **segmentation result of MRI with MMBs.

In Figure 6, we show how the active contour model works on the MRIs of phantom without and with MMBs, respectively. In our experiments, we fixed μ = 0, ν = 100 and λ_11 _= λ_22 _= 1. When MMBs are not used, 50 iterations and MRI segmentation are illustrated in Figures 6(a)&(b), respectively. When MMBs are used, 50 iterations and MRI segmentation are illustrated in Figures 6(c)&(d), respectively. Compared with Figures 6(b)&6(d), Figure 6(d) has better contrast and smoother outline, showing MRI using MMBs is more beneficial to object segmentation. However, the difference between Figure 5(d) and Figure 5(d') is far more than that between Figure 6(b) and Figure 6(d), which shows the improvements of US image segmentation with MMBs is much better than the improvements of MRI segmentation with MMBs. MMBs have greater influence on US imaging than on magnetic resonance imaging.

### US-MRI registration based on the proposed method

**Figure 7:**
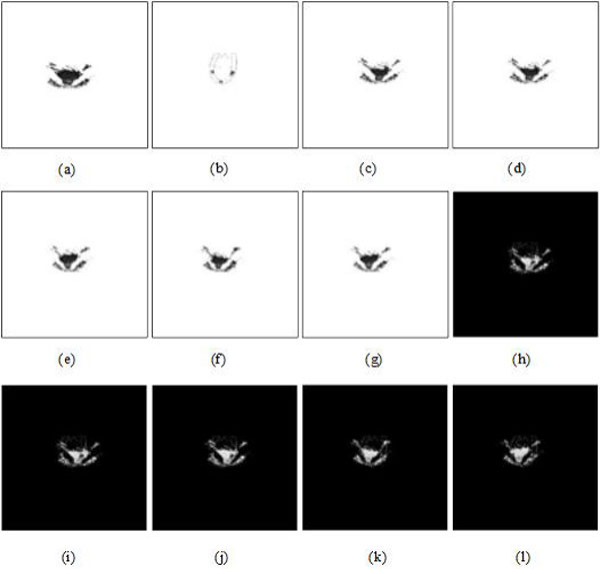
**US-MRI registration (from (c) to (g)) by different values of parameter *λ*_2 _(*λ*_1 _= 5, *λ*_3 _= 1) without MMBs based on the proposed methods**. **(a) **US image segmentation. **(b) **MRI segmentation. **(c) **(*λ*_2 _= 1*e *- 4). **(d) **(*λ*_2 _= 1*e *- 2). **(e) **(*λ*_2 _= 1). **(f) **(*λ*_2 _= 1*e *+ 2). **(g) **(*λ*_2 _= 1*e *+ 4). **(h) **subtraction result between (c) and (b). **(i) **subtraction result between (d) and (b). **(j) **subtraction result between (e) and (b). **(k) **subtraction result between (f) and (b). **(l) **subtraction result between (g) and (b).

**Figure 8:**
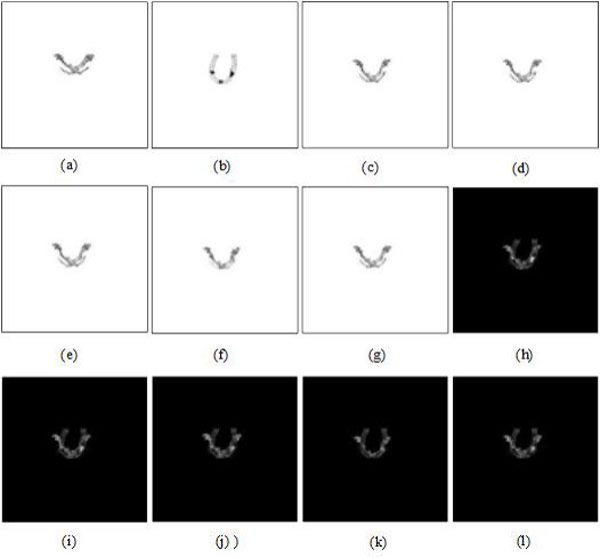
**US-MRI registration (from (c) to (g)) by different values of parameter *λ*_2 _(*λ*_1 _= 5, *λ*_3 _= 1) with MMBs based on the proposed methods**. **(a) **US image segmentation. **(b) **MRI segmentation. **(c)) **(*λ*_2 _= 1*e *- 4). **(d) **(*λ*_2 _= 1*e *- 2). **(e) **(*λ*_2 _= 1). **(f) **(*λ*_2 _= 1*e *+ 2). **(g) **(*λ*_2 _= 1*e *+ 4). **(h) **subtraction result between (c) and (b). **(i) **subtraction result between (d) and (b). **(j) **subtraction result between (e) and (b). **(k) **subtraction result between (f) and (b). **(l) **subtraction result between (g) and (b).

The registration results were compared by changing parameter λ_2 _with/without MMBs when λ_1 _and λ_3 _are fixed to 5 and 1, respectively, as shown in Figures 7 &8. According to Figures 7 &8, the results of root mean square error (RMS), peak signal to noise ratio (PSNR), correlation coefficient (COR), and mutual information (MI) were quantitatively analyzed and given in Table 1.

**Table 1:** Comparison of registration results (see Figures 7 & 8) by different values of parameter *λ*_2 _(*λ*_1 _= 5, *λ*_3 _= 1) based on the proposed method.

methods category		performance evaluation			
		
		RMS	PSNR	COR	MI
without MMBs	*λ*_2 _= 1e-4	0.0874	21.1311	0.3145	0.0605
	
	*λ*_2 _= 1e-2	0.0887	21.0074	0.3060	0.0618
	
	*λ*_2 _= 1	0.0915	20.7375	0.3111	0.0624
	
	*λ*_2 _= 1e+2	0.0843	21.4505	0.3100	0.0629
	
	*λ*_2 _= 1e+4	0.0852	21.3619	0.3075	0.0661

with MMBs	*λ*_2 _= 1e-4	0.0511	25.7932	0.4004	0.0811
	
	*λ*_2 _= 1e-2	0.0514	25.7391	0.3995	0.0806
	
	*λ*_2 _= 1	0.0515	25.7226	0.4090	0.0815
	
	*λ*_2 _= 1e+2	0.0435	27.1892	0.5303	0.0803
	
	*λ*_2 _= 1e+4	0.0483	26.2765	0.4545	0.0827

When the reference and the floating image (or the registered image), are compared, the RMS of the pairwise differences of the two images can serve as a measure how far on average the error is from 0. When RMS is small, the similarity between the two images is greater.

Peak signal-to-noise ratio, often abbreviated PSNR, is an engineering term for the ratio between the maximum possible power of a signal and the power of corrupting noise that affects the fidelity of its representation. This ratio can be used as a quality measurement between the reference and the registered image. The higher the PSNR, the better the quality of the registered image.

COR is a mathematical measure of how much one image can expect to be influenced by changes in another. It is closely related to covariance. If there is no relationship between the two images the COR is very low.

MI is a fundamental concept in information theory, and a measurement about statistical correlation of two random variables. Consider gray values of two images which will be registered as two random variables, when the both images achieve the best registration, MI approaches the maximum. When RMS after registration is smaller than before registration, and three indices (PSNR, COR and MI) after registration is larger than before registration, it is called the normal variation, otherwise called abnormal variation in the following discussion.

As shown in Table 1 we can achieve a better registration result independent of MMBs based on the proposed methods when λ_1_, λ_2 _and λ_3 _are set to 5, 1e+2 and 1, respectively.

**Figure 9:**
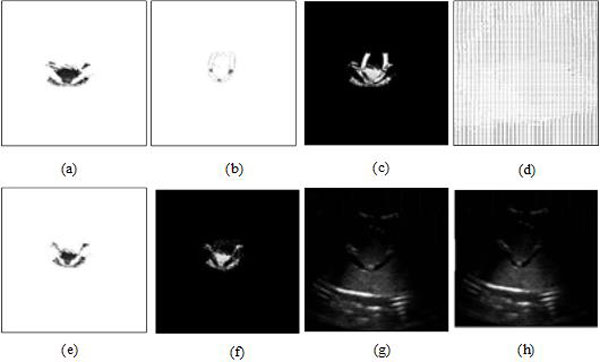
**US-MRI registration based on the proposed method without MMBs (*λ*_1 _= 5, *λ*_2 _= 1*e *+2, *λ*_3 _= 1)**. **(a) **US image segmentation. **(b) **MRI segmentation. **(c) **subtraction result between (a) and (b). **(d) **optical flow field. **(e) **registered result of (a). **(f) **subtraction result between (e) and (b). **(g) **US image. **(h) **registered US image.

**Figure 10:**
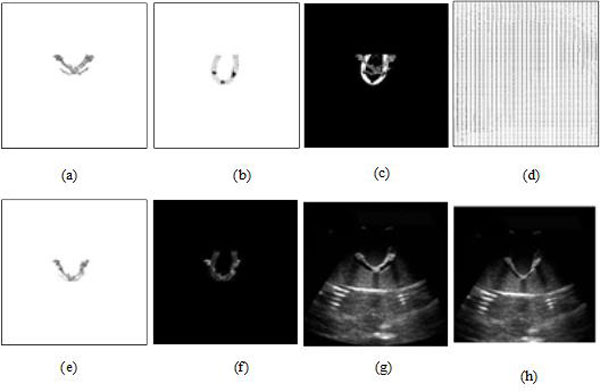
**US-MRI registration based on the proposed method with MMBs (*λ*_1 _= 5, *λ*_2 _= 1*e *+2, *λ*_3 _= 1)**. (a) US image segmentation. (b) MRI segmentation. (c) subtraction result between (a) and (b). (d) optical flow field. (e) registered result of (a). (f) subtraction result between (e) and (b). (g) US image. (h) registered US image.

Figures 9 &10 demonstrate US-MRI registration using the proposed method (λ_1 _= 5, λ_2 _= 1*e *+ 2, λ_3 _= 1) without and with MMBs, respectively. As it can be intuitively observed, Figure 10(f) has obvious advantages over Figure 9(f) from the perspective of registration. The four evaluation indexes (RMS, PSNR, COR and MI) in Table 2 had normal variations with and without MMBs, before and after registration, respectively. In addition, after registration, performance evaluation with the use of MMB had better improvement than that without MMB. For example, RMS decreases from 0.0843 to 0.0435, PSNR, COR and MI rises up from 21.4505 to 27.1892, from 0.3100 to 0.5303 and from 0.0629 to 0.0803, respectively. To sum up, the qualitative and quantitative analyses showed that US-MRI registration based on the proposed method is effective.

**Table 2:** Comparison of the registration results (see Figures 9 & 10) based on the proposed method (*λ*_1 _= 5, *λ*_2 _= 1e+2, *λ*_3 _= 1).

the proposed method		performance evaluation			
		
		RMS	PSNR	COR	MI
without MMBs	before registration	0.1148	18.7706	0.2027	0.0437
	
	after registration	0.0843	21.4505	0.3100	0.0629

with MMBs	before registration	0.0657	23.6063	0.1415	0.0398
	
	after registration	0.0435	27.1892	0.5303	0.0803

### US-MRI registration based on the other methods

**Figure 11:**
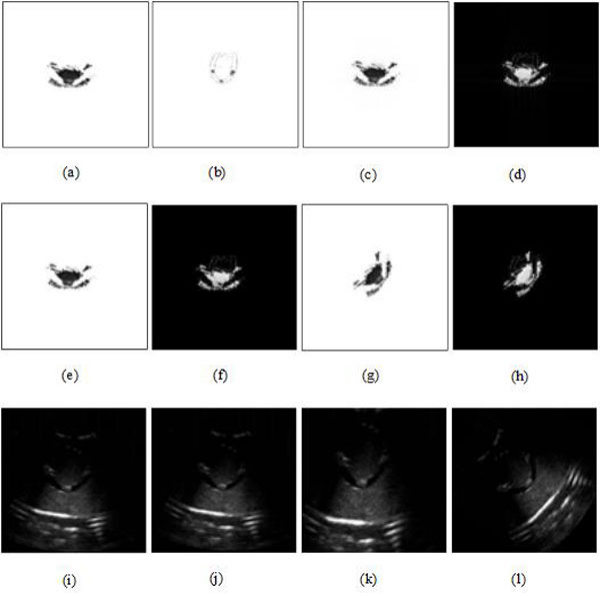
**US-MRI registration based on the other methods without MMBs**. **(a) **US imge Segmentation. **(b) **MRI segmentation. **(c) **registered result of (a) using FFT. **(d) **subtraction result between (c) and (b). **(e) **registered result of (a) using PSO. **(f) **subtraction result between (e) and (b). **(g) **registered result of (a) using MMI. **(h) **subtraction result between (g) and (b). **(i) **US image. **(j) **registered US image using FFT. **(k) **registered US image using PSO. **(l) **registered US image using MMI.

**Figure 12:**
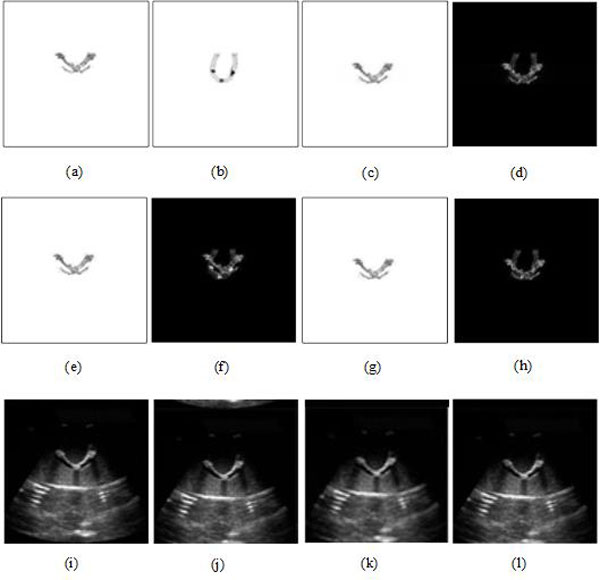
**US-MRI registration based on the other methods with MMBs**. **(a) **US imge segmentation. **(b) **MRI segmentation. **(c) **registered result of (a) using FFT. **(d) **subtraction result between (c) and (b). **(e) **registered result of (a) using PSO. **(f) **subtraction result between (e) and (b). **(g) **registered result of (a) using MMI. **(h) **subtraction result between (g) and (b). **(i) **US image. **(j) **registered US image using FFT. **(k) **registered US image using PSO. **(l) **registered US image using MMI.

Figures 11 &12 are US-MRI registration based on several other methods without and with MMBs, respectively. The other methods included fast Fourier transform (FFT) [27,28], particle swarm optimization (PSO) [29] and mutual information (MMI) [30,31]. From Table 2 without MMBs, before registration, RMS, PSNR, COR and MI are 0.1148, 18.7706, 0.2027 and 0.0437, respectively. After registration, for FFT and PSO, the evaluation indexes used in Table 3 were improved, but not much. For MMI, the increase of RMS (from 0.1148 to 0.1156), the decrease of PSNR (from 18.7706 to 18.7069) and the decrease of COR (from 0.2027 to 0.1494) are all abnormal variation as shown in Table 3. From Figure 11, it can be seen that the registration results using the other methods are unsatisfactory when MMBs are not used. The quantitative performance evaluated in Table 3 also confirms our intuitive feelings.

**Table 3:** Comparison of the registration results (see Figures 11 & 12) based on the other methods.

methods category		performance evaluation			
		
		RMS	PSNR	COR	MI
without MMBs	FFT	0.107	19.3786	0.2888	0.0658
	
	PSO	0.1139	18.8351	0.2338	0.0500
	
	MMI	0.1156	18.7069	0.1494	0.0526

with MMBs	FFT	0.0565	24.9184	0.3684	0.0736
	
	PSO	0.0652	23.6767	0.1629	0.0597
	
	MMI	0.0577	24.7385	0.3458	0.0697

In addition, with MMBs and before registration, Table 2 shows RMS, PSNR, COR and MI are 0.0657, 23.6063, 0.1415 and 0.0398, respectively. After registration, for FFT, PSO and MMI, the above evaluation indexes are improved.

The above quantitative analyses also indicate that FFT is the best method among the other three methods, which is in accordance with the above intuitive observation as shown in Figure 12. It should be noted that the evaluation indexes of the proposed method with MMBs, namely RMS, PSNR, COR and MI, are 0.0435, 27.1892, 0.5303 and 0.0803, while the evaluation indexes of FFT with MMB are 0.0565, 24.9184, 0.3684 and 0.0736. Clearly, the performance evaluation of the registration results using the proposed method are superior to the method with FFT. In summary, compared with the other methods, the proposed method combined with MMB has the best performance.

## Conclusions

In this study, MMBs were introduced as a new dual-modality contrast agent into the field of medical imaging. We verified that MMBs can increase the contrast of both US image and MRI, leading to the potential beneficial to registration of US and MR images. Using the same contrast agent for both US image and MRI would not only bring convenience to medical professionals, but also reduced health care cost. Qualitative and quantitative analyses of multiple group comparison experiments showed that registration results using all methods tested in this paper without MMBs were unsatisfactory. On the contrary, the proposed method combined with MMBs led to the best registration results.

Our algorithm implementation was intensity-based and was independent of the metric used. Therefore, it can be adapted to different image modalities. At present, effort to improve algorithms for medical image processing has seen very little progress. Combining novel nanomaterials with algorithm optimization provides a new approach for potential gains in imaging processing.

Our results were encouraging. However, they were still at preliminary stage. Further *in vivo *studies including toxicological and pathological studies will be necessary before our methods could be implemented in clinical applications.

## Competing interests

Other than the grants listed in the acknowledgement section, the authors declare that they have no other competing interest.

## Authors' contributions

NG, MH and FY were responsible for the design and overall investigation. MH, CC and SL were responsible for computational modeling and programming. DT helped to revise the manuscript. All authors 1) have made substantial contributions to conception and design, or acquisition of data, or analysis and interpretation of data; 2) have been involved in drafting the manuscript or revising it critically for important intellectual content; and 3) have given final approval of the version to be published. Each author has participated sufficiently in the work to take public responsibility for appropriate portions of the content.

## Authors' information

The Southeast University group (Mo Hou, Shouhua Luo, Fang Yang and Ning Gu) has been publishing in preparation and application of magnetic microbubbles, see website: http://lbmd.seu.edu.cn/research.php

Chen has been doing research in medical image processing, see website: http://cae.nuaa.edu.cn/swyx/webs/teachers/chencx.htm

Tang has been publishing image-based modeling work in recent years, For more information, please visit Tang's website: http://users.wpi.edu/~dtang/.

## Declarations

Publication of this article was paid with funding from the National Important Science Research Program of China (Grant No. 2013CB733804).

This article has been published as part of *BioMedical Engineering OnLine *Volume 14 Supplement 1, 2015: Cardiovascular Disease and Vulnerable Plaque Biomechanics. The full contents of the supplement are available online at http://www.biomedical-engineering-online.com/supplements/14/S1
